# Analyses of thymocyte commitment to regulatory T cell lineage in thymus of healthy subjects and patients with 22q11.2 deletion syndrome

**DOI:** 10.3389/fimmu.2023.1088059

**Published:** 2023-03-08

**Authors:** Simon Borna, Beruh Dejene, Uma Lakshmanan, Janika Schulze, Kenneth Weinberg, Rosa Bacchetta

**Affiliations:** ^1^ Division of Hematology, Oncology, Stem Cell Transplantation and Regenerative Medicine, Department of Pediatrics, Stanford University School of Medicine, Stanford, CA, United States; ^2^ Epimune GmbH, Berlin, Germany; ^3^ Center for Definitive and Curative Medicine (CDCM), Stanford University School of Medicine, Stanford, CA, United States

**Keywords:** Treg – regulatory T cell, 22q11.2DS, TSDR, Treg progenitors, FOXP3

## Abstract

The Chromosome 22q11.2 deletion syndrome (22q11.2DS) results in an inborn error of immunity due to defective thymic organogenesis. Immunological abnormalities in 22q11.2DS patients are thymic hypoplasia, reduced output of T lymphocytes by the thymus, immunodeficiency and increased incidence of autoimmunity. While the precise mechanism responsible for increased incidence of autoimmunity is not completely understood, a previous study suggested a defect in regulatory T cells (Treg) cell lineage commitment during T cell development in thymus. Here, we aimed to analyze this defect in more detail. Since Treg development in human is still ill-defined, we first analyzed where Treg lineage commitment occurs. We performed systematic epigenetic analyses of the Treg specific demethylation region (TSDR) of the *FOXP3* gene in sorted thymocytes at different developmental stages. We defined CD3+CD4+CD8+ FOXP3+CD25+ as the T cell developmental stage in human where TSDR demethylation first occurs. Using this knowledge, we analyzed the intrathymic defect in Treg development in 22q11.2DS patients by combination of TSDR, CD3, CD4, CD8 locus epigenetics and multicolor flow cytometry. Our data showed no significant differences in Treg cell frequencies nor in their basic phenotype. Collectively, these data suggest that although 22q11.2DS patients present with reduced thymic size and T cell output, the frequencies and the phenotype of Treg cell at each developmental stage are surprisingly well preserved.

## Introduction

At an estimated prevalence of 1:3000-1:7000 live births, the Chromosome 22q11.2 Deletion Syndrome (22q11.2DS) is one of the most common inborn errors of immunity (1). The underlying 1.5-3Mb interstitial deletions result in hemizygosity for ~40-100 different genes (2, 3). 22q11.2DS patients have significant phenotypic variation, with the most common manifestations being craniofacial anomalies, thymic hypoplasia, congenital heart disease (CHD) and hypoparathyroidism (4). In mice, hemizygosity for *Tbx1* and *Crkl* result in abnormal thymic organogenesis, suggesting that these are the two genes encoded at 22q11.2DS which account for the defective organogenesis and thymopoiesis in affected patients (5). Affected patients have reduced thymic size, and rarely, complete thymic absence, with concomitant reduced peripheral T cell numbers in early childhood. Over time, the T lymphopenia is partially compensated by peripheral T cell expansion, which likely explains the biased TCR repertoire with increased TCR clonality observed in these patients (6, 7). Interestingly, in addition to immunodeficiency, 22q11.2DS patients have a high incidence of autoimmunity, including but not limited to juvenile idiopathic arthritis (JIA), autoimmune thyroiditis, and immune cytopenias, e.g., thrombocytopenia, as well as allergy (8–11). The mechanisms responsible for the increased incidence of autoimmunity are not fully understood. A plausible explanation is that the defects in T cell development and selection, and/or reduced thymic output followed by peripheral T cell expansion may lead to escape and expansion of autoreactive T cells and aberrant B cell co-stimulation. In addition, Marcovecchio et al. showed that 22q11.2DS patients have reduced function of regulatory T (Treg) cells and decreased frequencies of CD4+FOXP3+ thymocytes compared to those of controls, suggesting dysfunction of 22q11.2DS Treg and possibly a thymic defect in Treg cell lineage commitment (12).

In this study, we analyzed the defect in Treg cell development in 22q11.2DS in more detail. Because the exact stage at which Treg cell lineage commitment occurs in human thymic development remains ill-defined, we first aimed to define the stage at which Treg commitment occurs, and then compared thymocytes from unaffected controls and 22q11.2DS patients.

In contrast to mice, where *Foxp3* expression is restricted to Treg cells, human *FOXP3* expression is also upregulated in conventional T (Tconv) cells after TCR receptor stimulation. Thus, *FOXP3* expression cannot be solely used as Treg cell lineage defining marker. Currently, the best way to define the Treg cell lineage in human is analysis of the Treg specific demethylation region (TSDR), a conserved non-coding regulatory element within the *FOXP3* gene (13). During thymic development, the TSDR is demethylated in response to TCR signaling, and together with multiple transcription factors and other epigenetic modifications, maintains high-level of *FOXP3* expression characteristic of Treg (14, 15). Therefore, we analyzed TSDR demethylation in thymocytes at different developmental stages by fluorescence activated cell sorting (FACS). We report that CD3+CD4+CD8+ CD25+FOXP3+ cells are the first intrathymic cell subset with fully demethylated TSDR, indicating that this is the first stage with detectable Treg cell lineage commitment. Next, we analyzed Treg cell development in control and 22q11.2DS patients, using TSDR, CD3, CD4 and CD8 locus epigenetic analyses and multicolor flow cytometry. In contrast to the previous report, we did not observe significant differences in Treg differentiation between control and 22q11.2DS patients. Collectively, these data suggest that although 22q11.2DS patients present with reduced thymus size and T cell output, the frequencies and basic phenotype of Treg cells at each developmental stage are surprisingly well preserved.

## Material and methods

### Patient and control sample collection and processing

Thymic tissue was obtained from children aged 2-15 months undergoing corrective surgery for CHD, in accordance with a protocol approved by the Stanford Institutional Review Board (IRB-16877). 22q11.2DS was diagnosed by routine screening by Fluorescence *in situ* hybridization (FISH) and/or comparative genomic hybridization (CGH) arrays. To control for possible physiologic effects of CHD on thymic development, the patients in both the control and 22q11.2DS groups had Tetralogy of Fallot with pulmonary atresia. Syndromic causes of CHD, e.g., trisomy 21 or Alagille syndrome, were excluded from the Control group. In total, 6 22q11.2DS patients and 10 control thymic samples from 2–15 month-old subjects were analyzed. For one control sample used only in TSDR demethylation analyses in CD25-FOXP3-, CD25+FOXP3- and CD25+FOXP3+ subpopulations of DP, and CD25+FOXP3+ SP thymocytes from control thymocytes, the exact age and type of heart defect are not specified. Given the very young age of the subjects, the 22q11.2DS patients’ samples were not selected based on clinical signs of autoimmunity. Thymic tissue was processed as described previously (16). Briefly, after removing the blood vessels, fat and connective tissues, the organ was cut into small pieces. The pieces were gently pressed by syringe plug and the supernatant containing thymocytes was collected. The pieces of tissue were transferred to RPMI supplemented with 100ug/mL DNAse (Sigma-Aldrich) and 100ug/mL Liberase (Sigma-Aldrich) and further dissociated using gentleMACS Dissociator (Miltenyi Biotec). Samples were incubated in 37°C for 20 min and the non-stromal cells in the supernatant were collected. The cells were pooled and either cryopreserved or used immediately as indicated below.

### Flow cytometry analyses of Treg cell development

At the day of the experiment, cryopreserved samples were thawed in RPMI media supplemented with 30% FBS, 10ug/mL DNAse I (Stemcell Technologies). Cells were rested for 20 min in 37°C, spun and stained on ice with LIVE/DEAD™ Fixable Violet, washed with PBS 2% FBS and stained for surface markers with CD3 BV785 [OKT3] (Biolegend), CD4 APC-R700 [RPA-T4] (BD), CD8 PE-Cy7 [SK1] (BioLegend), CD25 APC [2A3] (BD), CD69 BUV395 [FN50] (BD), CD1a BUV496 [HI149] (BD), CD14 PB [63D3] (BioLegend) antibodies in PBS supplemented with 1% BSA and brilliant stain buffer (BD) for 30 min on ice. Cells were washed, fixed and permeabilized using Foxp3/Transcription Factor Staining Buffer Set (Invitrogen) and stained with FOXP3 A488 [259D] (BioLegend) and FOXP3 PE [150D] (BioLegend) antibodies or isotype controls 488 and PE [MOPC-21] (BioLegend). Cells were washed and analyzed using BD FACSymphony™ A5.

### Cell sorting and epigenetic analyses

For TSDR demethylation analyses in CD25-FOXP3-, CD25+FOXP3- and CD25+FOXP3+ subpopulations of DP, and CD25+FOXP3+ SP thymocytes from control thymocytes, we enriched CD25+ cells from fresh thymocytes using CD25 MicroBeads II (Miltenyi Biotec). Positive and negative fraction were stained separately or mixed together in 1:1 cell ratio and stained for surface markers with CD3 PE [OKT3] (BioLegend), CD4 A700 [RPA-T4] (BioLegend), CD8 PE-Cy7 [SK1], CD25 APC [2A3] (BD) antibodies. Cells were fixed and permeabilized using Foxp3/Transcription Factor Staining Buffer Set (Invitrogen), stained with FOXP3 A488 [259D] (Biolegend) and sorted on BD FACS Aria instrument. Sorted cell pellets were frozen at -80°C in PBS.

TSDR, CD3, CD4 and CD8 locus methylation analyses in controls and 22q11.2DS patients was done from frozen thymocytes. Cells were thawed in RPMI supplemented with 30% FBS, 1% penstrep, 10ug/mL of DNAseI (Merck), heparin 20U/mL (17) and rested for 1 hour in 37°C. Cells were stained in PBS 2%FBS with CD3 PerCyp5.5 [SK7] (BioLegend), CD4 FITC [SK3] (BioLegend), CD8 BV421 [RPA-T8] (BD) antibodies for 30 min on ice. After wash, each thymocyte population was sorted in duplicate into RPMI 10% FBS. Sorted cell pellets were spun down and washed with PBS.

TSDR, CD3, CD4 and CD8 locus methylation analyses were performed as described previously (18). Briefly, upon thaw, genomic DNA was isolated, bisulfite converted and subjected to qPCR using methylation specific primers.

### Statistical analyses

Statistical significance was evaluated using GraphPad software (Dotmatics). Multiple comparisons were done using multiple measure ANOVA, when data represented matching values from the same donor, or regular ANOVA, when some samples were missing. The differences between the control group and the experimental groups were done using Dunnett’s posttest. The significance between the 22q11.2DS and control samples was evaluated using t-test. Star symbols in figures represent p values from following ranges *p<0.05, **p<0.01, ***p<0.005,****p<0.0001. The non-significant comparisons are not indicated.

## Results

### The expression of FOXP3 and CD25, and the TSDR demethylation occur simultaneously during T cell development in mature double positive stage

**Figure 1:**
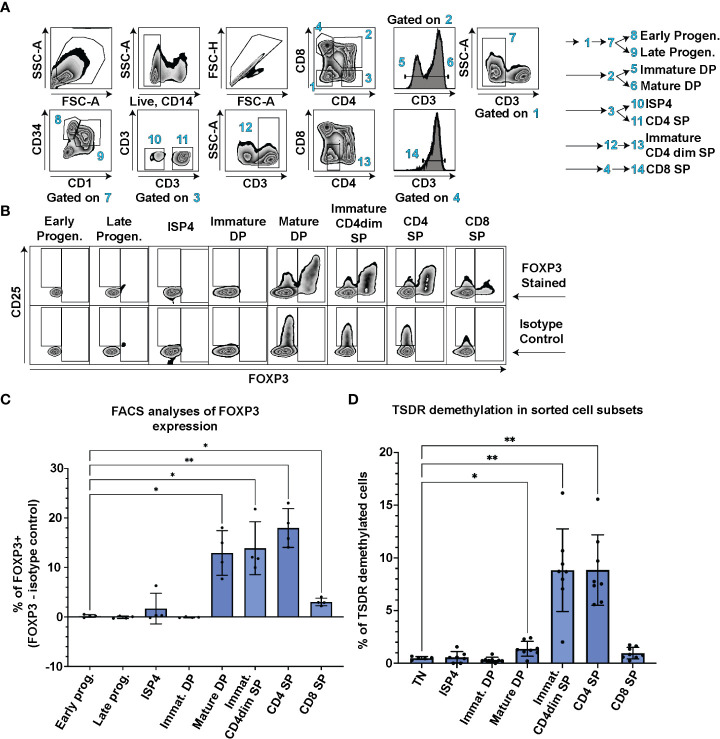
TSDR demethylation, FOXP3 and CD25 expression analyses at different stages of T cell development. **(A)** FACS gating strategy to identify CD3-CD4-CD8-CD34+CD1- Early progenitors, CD3-CD4-CD8-CD34+CD1+ Late progenitors, CD3-CD4+CD8- ISP4, CD3-CD4+CD8+ immature DP, CD3+CD4+CD8+ mature DP, CD3+CD4dimCD8- Immature CD4dim SP, CD3+CD4+CD8- SP CD4 and CD3+CD4-CD8+ SP CD8 cell populations. Data show representative FACS plots of a control thymocytes. **(B)** FOXP3 and CD25 expression in thymocyte subsets described in **(A)**. The top row shows representative control thymocytes subsets stained with anti FOXP3 antibody and the bottom row the same donor’s cell subsets stained with isotype control antibody. **(C)** Quantification of FOXP3 expression in cell populations gated as in A and **(B)** % of isotype control positive cells were subtracted from % of FOXP3+ cells to control for differences in non-specific binding of the FOXP3 anybody to each of the cell population. Data were analyzed using one way analyses of variants repeated measures ANOVA and Dunnett’s multiple comparisons test (n=4), and only significant differences are depicted in the graph. **(D)** TSDR demethylation analyses of sorted thymocyte populations. Each cell population was sorted in technical duplicate and averages from the TSDR demethylation analyses is plotted. Gating was similar as in **(A)** In addition, CD3-CD4-CD8- TN cells were added to the analyses. Data were analyzed using Brown-Forsythe ANOVA test and Dunnett’s T3 multiple comparison test (n=8), and only significant differences are depicted in the graph. Star symbols in figures represent p values from following ranges *p < 0.05, **p < 0.01.

First, we aimed to determine when Treg cell lineage commitment occurs during T cell development. We probed FOXP3 expression in CD3-CD4-CD8-CD34+CD1- early and CD3-CD4-CD8-CD34+CD1+ late thymic progenitors, CD3-CD4+CD8- immature single positive cells (ISP4), CD3-CD4+CD8+ immature and CD3+CD4+CD8+ mature double positive (DP), CD3+CD4dimCD8- immature single positive (SP) CD4dim, and CD3+CD4+CD8- SP CD4 and CD3+CD4-CD8+ SP CD8 thymocytes using FACS in thymocytes from 4 controls (**Figure 1A**). In parallel, we stained the samples with isotype control antibody and subtracted the number of isotype control positive cells from the FOXP3 positive cells to correct for any artefacts resulting from differences in nonspecific binding of the antibodies to each cell population. The earliest stage of thymic differentiation expressing FOXP3 was the CD3+CD4+CD8+ mature DP (**Figures 1B, C**). Interestingly, the mature DP thymocytes were also the first population containing CD25 high cells (**Figure 1B**).

In contrast to mice, human T conventional cells express FOXP3 upon TCR stimulation and thus FOXP3 expression cannot be solely used as a Treg lineage defining marker. Therefore, we sorted all the main thymocyte populations and performed analysis of TSDR demethylation using methylation sensitive qPCR. This method enumerates the frequency of cells with fully/largely demethylated TSDR from given sample (18). We analyzed TSDR demethylation in sorted CD3-CD4+CD8- ISP4, CD3-CD4+CD8+ immature and CD3+CD4+CD8+ mature DP, CD3+CD4dimCD8- immature SP CD4dim, and CD3+CD4+CD8- SP CD4 and CD3+CD4-CD8+ SP CD8 thymocytes. As a negative control, we sorted CD3-CD4-CD8- triple negative cells (TN). In line with the detectable FOXP3 expression, we observed significant increase in frequency of TSDR demethylated cells at the mature DP stage compared to TN (**Figure 1D**). Together, these data demonstrate that FOXP3 and CD25 expression, and TSDR demethylation occur first during T cell development at the mature DP stage.

### CD3+CD4+CD8+FOXP3+CD25+ mature DP thymocytes represent the first population fully committed to the Treg lineage in human thymus

**Figure 2:**
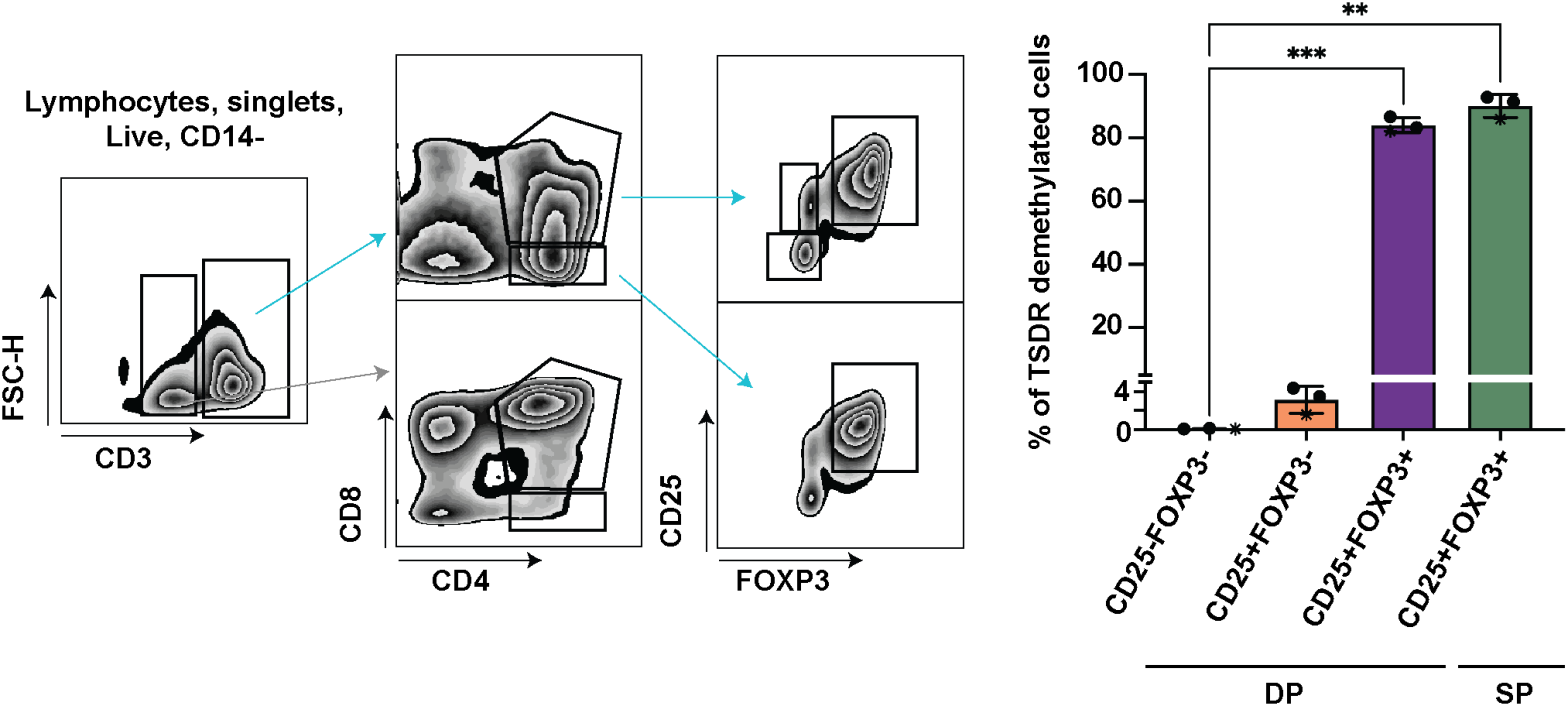
TSDR demethylation analyses in CD25-FOXP3-, CD25+FOXP3- and CD25+FOXP3+ subpopulations of DP, and CD25+FOXP3+ SP thymocytes. Sorting strategy shown on left and quantification of TSDR demethylation analyses on right. The sorting strategy shows CD25 enriched cells mixed in approximately 1:1 ratio with CD25- fraction (datapoints shown on right in stars). In the two other experiments, the respective cell subsets were sorted separately from CD25+ and CD25- cell populations. The CD3- cells (gray arrow) are shown to give the reader a better information about the gating strategy. Data were analyzed using one-way analyses of variants repeated measures ANOVA and Dunnett’s multiple comparisons test (n=3), and only significant differences are depicted in the graph. Star symbols in figures represent p values from following ranges *p < 0.05, **p < 0.01, ***p < 0.005.

To dissect which cells are responsible for the increase of TSDR demethylation in the mature DP stage, we further divided mature DP cells into 3 subsets CD25-FOXP3-, CD25+FOXP3- and CD25+FOXP3+, and sorted these cells for TSDR demethylation analysis. As a positive control of TSDR demethylation, we sorted FOXP3+ CD25+ SP CD4 cells (**Figure 2**). To obtain sufficient numbers of cells for the TSDR demethylation analysis, we enriched CD25 cells from fresh thymocytes and sorted CD25+ populations from the enriched cells and CD25- cells from the corresponding negative fraction. We found TSDR demethylated cells almost exclusively within the FOXP3+CD25+ population and only a very low percentage of TSDR demethylated cells among CD25+FOXP3- cells. To reduce possible contamination of CD25+FOXP3- cells by TSDR demethylated cells, which may result from FACS sort impurity, we mixed the CD25- fraction with CD25+ fraction obtained after CD25 bead enrichment. This adjustment of the experimental design makes the cell impurities from FACS-sort more likely to originate from cells, which are CD25 low and thus likely TSDR methylated. This adjustment further reduced the fraction of TSDR demethylated cells in CD25+FOXP3- cells (**Figure 2**, star datapoints). Together with the above-described data, we demonstrated that TSDR demethylation occurs for the first-time during T cell development concomitantly with FOXP3 expression, but that CD25 expression is not exclusively linked with the TSDR demethylation. Considering TSDR demethylation as a definitive marker of Treg cell lineage commitment, then fully committed Treg cells appear for the first time at the DP stage in CD25+FOXP3+ cells.

### Demethylation state of TSDR, CD3, CD4 and CD8 loci in thymocytes from 22q11.2DS patients is largely preserved

**Figure 3:**
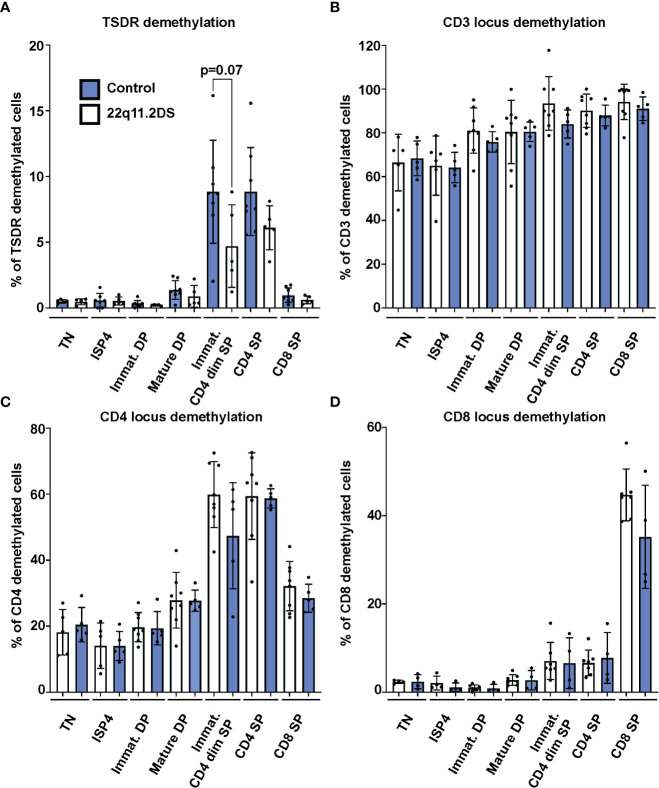
TSDR **(A)**, CD3 **(B)**, CD4 **(C)** and CD8 **(D)** locus demethylation analyses in sorted thymocytes subpopulations from 8 control (blue) and 5 22q11.2DS patients (white). Significance was evaluated using student t-test. The data have not reached significance and only trend toward lower % of TSDR demethylated cells in immature CD4dim SP cells is shown.

Next, we analyzed frequencies of cells with TSDR, CD3, CD4 and CD8 locus demethylation in sorted CD3-CD4-CD8- TN, CD3-CD4+CD8- ISP4, CD3-CD4+CD8+ immature and CD3+CD4+CD8+ mature DP, CD3+CD4dimCD8- immature SP CD4dim, and CD3+CD4+CD8- SP CD4 and CD3+CD4-CD8+ SP CD8 thymocytes from eight controls and five 22q11.2DS patients. Results showed no significant differences in TSDR demethylation in any of the subpopulation tested. However, we observed a trend toward lower frequency of TSDR demethylated cell in immature SP CD4dim cells of 22q11.2DS patients compared to controls (**Figure 3A**). Although we did not detect any difference in either CD3, CD4 or CD8 locus demethylation (**Figures 3B–D**), there was a tendency toward lower frequency of CD4 locus demethylated cells in immature SP CD4dim cells and lower in frequency of CD8 locus demethylated cells in CD8 SP cells. However, the tendencies were rather very subtle and thus, to reach a definitive conclusion about its significance, a larger cohort would have to be analyzed. Interestingly, we observed high frequency of cells with demethylated CD3 loci, intergenic *CD3G* and *CD3D* region specifically demethylated in CD3 T cells (18), in TN thymocytes. This is an interesting phenomenon, which may be useful as a tool to identify an early T cell progenitor. In summary, we did not observe any major differences in frequency of TSDR demethylated cells, suggesting largely preserved mechanisms behind Treg cell lineage commitment in 22q11.2DS patients.

### 22q11.2DS Treg cells are present in normal frequencies in each developmental stage and present with normal expression levels of FOXP3 isoforms, CD25, CD3 and the activation marker CD69

**Figure 4:**
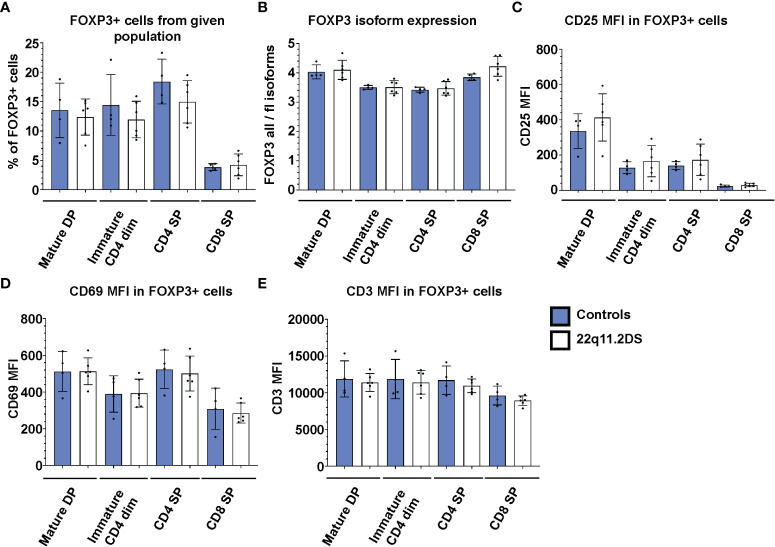
FACS analyses of FOXP3 positive cells in thymocyte subsets of 4 control (blue) and 6 22q11.2DS (white) patients. The data show **(A)** % of FOXP3+ cells **(B)** median fluorescent intensity (MFI) from ratio of all FOXP3 isoforms to FOXP3 full length isoforms in FOXP3+ cells, **(C)** MFI of CD25, **(D)** CD69 and **(E)** CD3 in FOXP3+ cells gated from each population as indicated in the graph. The statistical significance was evaluated using student t-test. None of the comparisons reached statistical significance.

Because the vast majority of the FOXP3+ cells in both DP and SP cells are TSDR demethylated (**Figure 2**), FOXP3 expression in the thymus can be used as a marker of bona fide Treg cells. To complement the epigenetic analyses, we analyzed the frequency and phenotype of FOXP3 positive cells in thymocytes from four controls and six 22q11.2DS patients to unravel previously suggested abnormalities in Treg cell lineage commitment in 22q11.2DS patients. First, we analyzed frequencies of FOXP3+ cells in each population with detectable FOXP3 expression, including mature DP, immature SP CD4dim, SP CD4 and SP CD8 (**Figures 1A–C**). Interestingly, we did not find any differences in the frequency of FOXP3+ cells in 22q11.2DS patients in any of the populations tested (**Figure 4A**). Consistent with the TSDR demethylation analyses, we observed a mild trend toward reduced numbers of FOXP3 positive cells in CD4 SP cell population, which did not achieve statistical significance (p=0.1841). Two major isoforms of FOXP3 are expressed in humans, the FOXP3 full length (flFOXP3) transcript and a shorter isoform missing exon 2 (FOXP3). An abnormal ratio of these isoforms is associated with aberrant Treg cell function in several autoimmune diseases (19). Therefore, we additionally tested the expression of these isoforms using two clones of antibodies, one of which recognizes all FOXP3 isoforms (259D), and the second which specifically binds to exon 2 (150D). We did not find any differences in expression of the flFOXP3 and FOXP3 isoforms between control and 22q11.2DS FOXP3+ thymocytes (**Figure 4B**). In addition, we measured CD25 and CD69 expression, which are upregulated upon TCR signaling. No differences in expression of either CD25 or CD69 in any of the FOXP3 positive population tested (**Figures 4C, D**). Similarly, we did not find differences in expression of CD3, the signaling subunit of TCR receptor, suggesting normal surface expression of TCR in developing Treg cells in patients with 22q11.2DS (**Figure 4E**). Collectively, we found that the frequencies and/or the expression of CD25, CD69 or CD3 in FOXP3+ cells at any stage of T cell development are comparable in 22q11.2DS patients and controls, suggesting largely preserved Treg cell lineage development and commitment in 22q11.2DS patients.

## Discussion

Here, we performed a systematic analysis of FOXP3 and CD25 expression, and TSDR demethylation at different stages of human T cell development and identify CD25+FOXP3+ cells among the DP thymocytes as the first population of fully committed Treg cells. These data are in line and further extend the pioneering work of Vanhanen et al. demonstrating the presence of TSDR demethylated cells among the human CD25+ DP thymocytes (20). Interestingly, we found only negligible frequencies of TSDR demethylated cells in CD25+FOXP3- DP thymocytes. Nevertheless, it is possible that some of CD25+FOXP3- thymocytes still might commit to Treg cell lineage, demethylate TSDR and express FOXP3. Consistently, both CD25+FOXP3- and FOXP3+ Treg progenitors have been identified among murine CD4 SP thymocytes (21) and two types of Treg-like cells with distinct transcriptional signatures have been identified among CD4 SP human thymocytes (22), suggesting that two independent Treg developmental pathways may exist also in humans. Overall, our data indicate that TSDR demethylation occurs simultaneously or after FOXP3 expression during intrathymic Treg development. Thus, DP thymocytes are the first stage at which fully committed Treg cells are detectable in the human thymus. The determination of the ontogeny of human Treg provides an indispensable knowledge for future work aiming to unravel defects in Treg cell lineage commitment in human diseases.

Towards this purpose, we aimed to better determine the defect in Treg cell lineage commitment in patients with 22q11.2DS. Analyses of peripheral blood populations in patients with 22q11.2DS have demonstrated decreased absolute numbers of FOXP3+ Treg (23–25), which has been hypothesized to contribute to the increased incidence of autoimmunity and allergies which affect a significant proportion of these patients. Our quantitative analyses of the early Treg commitment from thymi of patients with 22q11.DS showed no statistically significant differences between patients and controls. This finding is in contrast to previously reported data by Marcovecchio et al, which might be due to a combination of the high variability of Treg frequencies in 22q11.2DS patients and a small 22q11.2DS patient sample size analyzed by Marcovecchio et al. (12). On the other hand, we saw a trend toward reduced Treg cell lineage commitment in CD4 single-positive stage thymocytes by both TSDR demethylation and FOXP3 expression analyses, suggesting that there might be a very subtle defect. Because the peripheral T lymphocyte pool represents the accumulation of thymic output over time, even subtle decreases in Treg commitment could result in a clinically significant paucity of mature Treg. However, consistently with the absence or negligible defect of Treg cell commitment, CD4+/Treg ratio in 22q11.2DS pediatric patients is normal (24, 25), indicating that thymic output accounts for a normal ratio of Treg and CD4+ Tconv cells.

Collectively, these data suggest normal Treg cell lineage commitment during T cell development in patients with 22q11.2DS and warrant additional studies of the mechanisms responsible for their increased incidence of autoimmunity. These studies will be crucial to determine appropriate therapeutic approaches to restore tolerance. For example, it is plausible to speculate that a divergent evolution of TCR repertoires during peripheral expansion of Treg and Tconv cells allows autoreactive T and/or B cells to escape the Treg dependent mechanisms of peripheral tolerance (26). In addition, reduced Treg cell suppressive function was reported in 22q11.2DS patients (12), but the molecular mechanism responsible for the reduced Treg suppressive function remains to be determined. In our work, we focused on evaluating the Treg lineage commitment during development in the thymus in controls and 22q11.2DS patients, and thus we have not analyzed the function of 22q11 Treg, which represents a limitation of this work and warrants future in-depth analyses of 22q11.2DS thymic and peripheral Treg function. Autologous engineered Treg therapy obtained from converted CD4+ Tconv cells might be a suitable option for 22q11.2DS patients (27), as such cells could overcome 22q11.2DS developmental, or cell intrinsic Treg defects as well as defects caused by divergent TCR evolution associated with peripheral T cell expansion.

## Data availability statement

The raw data supporting the conclusions of this article will be made available by the authors, without undue reservation.

## Ethics statement

The studies involving human participants were reviewed and approved by Stanford Institutional Review Board (IRB-16877). Written informed consent to participate in this study was provided by the participants’ legal guardian/next of kin.

## Author contributions

Leading of the project: RB and KW. Sample processing and data analyzes: SB, UL, BD, and JS. Funding acquisition: RB and KW. Supervision: RB and KW. Writing original draft: SB, RB, and KW. Review & editing: SB, UL, BD, RB, JS, and KW. All authors contributed to the article and approved the submitted version.

## References

[1] Kobrynski LJ Sullivan KE. Velocardiofacial syndrome, digeorge syndrome: The chromosome 22q11.2 deletion syndromes. Lancet (2007) 370(9596):1443–52. doi: 10.1016/S0140-6736(07)61601-8 17950858

[2] Du Q de la Morena MT van Oers NSC. The genetics and epigenetics of 22q11.2 deletion syndrome. Front Genet (2019) 10:1365. doi: 10.3389/fgene.2019.01365 32117416 PMC7016268

[3] Guna A Butcher NJ Bassett AS. Comparative mapping of the 22q11.2 deletion region and the potential of simple model organisms. J Neurodev Disord (2015) 7(1):18. doi: 10.1186/s11689-015-9113-x 26137170 PMC4487986

[4] Cirillo A Lioncino M Maratea A Passariello A Fusco A Fratta F. Clinical manifestations of 22q11.2 deletion syndrome. Heart Fail Clin (2022) 18(1):155–64. doi: 10.1016/j.hfc.2021.07.009 34776076

[5] Guris DL Duester G Papaioannou VE Imamoto A. Dose-dependent interaction of Tbx1 and crkl and locally aberrant Ra signaling in a model of Del22q11 syndrome. Dev Cell (2006) 10(1):81–92. doi: 10.1016/j.devcel.2005.12.002 16399080

[6] Piliero LM Sanford AN McDonald-McGinn DM Zackai EH Sullivan KE. T-Cell homeostasis in humans with thymic hypoplasia due to chromosome 22q11.2 deletion syndrome. Blood (2004) 103(3):1020–5. doi: 10.1182/blood-2003-08-2824 14525774

[7] Pierdominici M Mazzetta F Caprini E Marziali M Digilio MC Marino B. Biased T-cell receptor repertoires in patients with chromosome 22q11.2 deletion syndrome (Digeorge Syndrome/Velocardiofacial syndrome). Clin Exp Immunol (2003) 132(2):323–31. doi: 10.1046/j.1365-2249.2003.02134.x PMC1808695 12699424

[8] Gennery AR Barge D O'Sullivan JJ Flood TJ Abinun M Cant AJ. Antibody deficiency and autoimmunity in 22q11.2 deletion syndrome. Arch Dis Child (2002) 86(6):422–5. doi: 10.1136/adc.86.6.422 PMC1763000 12023174

[9] Davies K Stiehm ER Woo P Murray KJ. Juvenile idiopathic polyarticular arthritis and iga deficiency in the 22q11 deletion syndrome. J Rheumatol (2001) 28(10):2326–34. 11669177

[10] Morsheimer M Brown Whitehorn TF Heimall J Sullivan KE. The immune deficiency of chromosome 22q11.2 deletion syndrome. Am J Med Genet A (2017) 173(9):2366–72. doi: 10.1002/ajmg.a.38319 28627729

[11] Jawad AF McDonald-Mcginn DM Zackai E Sullivan KE. Immunologic features of chromosome 22q11.2 deletion syndrome (Digeorge Syndrome/Velocardiofacial syndrome). J Pediatr (2001) 139(5):715–23. doi: 10.1067/mpd.2001.118534 11713452

[12] Marcovecchio GE Bortolomai I Ferrua F Fontana E Imberti L Conforti E. Thymic epithelium abnormalities in digeorge and down syndrome patients contribute to dysregulation in T cell development. Front Immunol (2019) 10:447. doi: 10.3389/fimmu.2019.00447 30949166 PMC6436073

[13] Baron U Floess S Wieczorek G Baumann K Grutzkau A Dong J. DNA Demethylation in the human Foxp3 locus discriminates regulatory T cells from activated Foxp3(+) conventional T cells. Eur J Immunol (2007) 37(9):2378–89. doi: 10.1002/eji.200737594 17694575

[14] Ohkura N Hamaguchi M Morikawa H Sugimura K Tanaka A Ito Y. T Cell receptor stimulation-induced epigenetic changes and Foxp3 expression are independent and complementary events required for treg cell development. Immunity (2012) 37(5):785–99. doi: 10.1016/j.immuni.2012.09.010 23123060

[15] Zheng Y Josefowicz S Chaudhry A Peng XP Forbush K Rudensky AY. Role of conserved non-coding DNA elements in the Foxp3 gene in regulatory T-cell fate. Nature (2010) 463(7282):808–12. doi: 10.1038/nature08750 PMC2884187 20072126

[16] Stoeckle C Rota IA Tolosa E Haller C Melms A Adamopoulou E. Isolation of myeloid dendritic cells and epithelial cells from human thymus. J Vis Exp (2013) 79):e50951. doi: 10.3791/50951 PMC3923894 24084687

[17] Denning SM Tuck DT Singer KH Haynes BF. Human thymic epithelial cells function as accessory cells for autologous mature thymocyte activation. J Immunol (1987) 138(3):680–6. doi: 10.4049/jimmunol.138.3.680 3492529

[18] Baron U Werner J Schildknecht K Schulze JJ Mulu A Liebert UG. Epigenetic immune cell counting in human blood samples for immunodiagnostics. Sci Transl Med (2018) 10(452). doi: 10.1126/scitranslmed.aan3508 30068569

[19] Mailer RKW. Alternative splicing of Foxp3-virtue and vice. Front Immunol (2018) 9:530. doi: 10.3389/fimmu.2018.00530 29593749 PMC5859138

[20] Vanhanen R Leskinen K Mattila IP Saavalainen P Arstila TP. Epigenetic and transcriptional analysis supports human regulatory T cell commitment at the Cd4+Cd8+ thymocyte stage. Cell Immunol (2020) 347:104026. doi: 10.1016/j.cellimm.2019.104026 31843201

[21] Owen DL Mahmud SA Sjaastad LE Williams JB Spanier JA Simeonov DR. Thymic regulatory T cells arise *Via* two distinct developmental programs. Nat Immunol (2019) 20(2):195–205. doi: 10.1038/s41590-018-0289-6 30643267 PMC6650268

[22] Park JE Botting RA Dominguez Conde C Popescu DM Lavaert M Kunz DJ. A cell atlas of human thymic development defines T cell repertoire formation. Science (2020) 367(6480). doi: 10.1126/science.aay3224 PMC7611066 32079746

[23] Ferrando-Martinez S Lorente R Gurbindo D De Jose MI Leal M Munoz-Fernandez MA. Low thymic output, peripheral homeostasis deregulation, and hastened regulatory T cells differentiation in children with 22q11.2 deletion syndrome. J Pediatr (2014) 164(4):882–9. doi: 10.1016/j.jpeds.2013.12.013 24461789

[24] Klocperk A Grecova J Sismova K Kayserova J Fronkova E Sediva A. Helios Expression in T-regulatory cells in patients with di George syndrome. J Clin Immunol (2014) 34(7):864–70. doi: 10.1007/s10875-014-0071-y 25008482

[25] Di Cesare S Puliafito P Ariganello P Marcovecchio GE Mandolesi M Capolino R. Autoimmunity and regulatory T cells in 22q11.2 deletion syndrome patients. Pediatr Allergy Immunol (2015) 26(6):591–4. doi: 10.1111/pai.12420 26058917

[26] Sng J Ayoglu B Chen JW Schickel JN Ferre EMN Glauzy S. Aire expression controls the peripheral selection of autoreactive b cells. Sci Immunol (2019) 4(34). doi: 10.1126/sciimmunol.aav6778 PMC7257641 30979797

[27] Borna S Lee E Sato Y Bacchetta R. Towards gene therapy for ipex syndrome. Eur J Immunol (2022) 52(5):705–16. doi: 10.1002/eji.202149210 PMC9322407 35355253

